# Alterations of Blood-Brain Barrier and Associated Factors in Acute Liver Failure

**DOI:** 10.1155/2013/841707

**Published:** 2013-05-26

**Authors:** Wei Cui, Cui-Ming Sun, Pei Liu

**Affiliations:** Department of Infectious Diseases, The First Affiliated Hospital, China Medical University, Shenyang 110001, China

## Abstract

Brain edema in acute liver failure (ALF) remains lethal. Cytotoxic mechanisms associated with brain edema have been well recognized, but the role of vasogenic mechanisms of brain edema has not been explored. Intact tight junctions (TJs) between brain capillary endothelial cells are critical for normal BBB function. Recent reports found significant alterations in the tight junction elements including occludin and claudin-5, suggesting a vasogenic injury in the blood-brain barrier (BBB) integrity. However, the role of TJ in ALF has not been completely understood. This paper reviews the role of the paracellular tight junction in the increased selective BBB permeability that leads to brain edema in ALF and furthermore explores the effect of systemic inflammatory cytokines on the tight junction dysfunction.

## 1. Introduction

Acute liver failure (ALF) is a clinical syndrome associated with massive hepatocellular necrosis and severe liver dysfunction in the absence of previous liver disease. Death occurs in over 70% of cases if without orthotopic liver transplantation. Brain edema and sepsis are the two leading causes of death [1]. It showed in patients with hepatic failure that incidence of brain edema reached 51.4% with autopsy, whereas it was 30.1% with clinical examination. When encephalopathy and brain edema occur, the disease takes a rapidly progressive and lethal course [2, 3].

The mechanisms responsible for the development of brain edema in ALF remain inadequately characterized. Klatzo categorized the mechanism of brain edema in general as being either cytotoxic, “intracellular swelling without increased permeability of BBB,” or vasogenic, “increased permeability of BBB leading to net gain of fluid,” with water and plasma constituents accumulated in the extracellular region as a consequence of structural BBB injury [4, 5].

In 1992, Kato and colleagues demonstrated swollen astrocytes and their end foot (markers of cytotoxic effects) but observed only minimal ultrastructural alterations in brain capillaries of patients who died of ALF. In fact, the brain capillary endothelial cell and its tight junction were relatively intact [6]. Similar findings were observed in animal models of ALF [7, 8]. Since there is no gross structural damage seen in the brain capillary membrane, the concept of vasogenic brain edema became unpopular, and for many years a cytotoxic event was considered to be the main mechanism in the pathogenesis of brain edema in ALF. The accepted tenet of cytotoxic brain edema in ALF has been recently challenged [9, 10].

Recent reports have not only found significant alterations in the tight junction elements including occludin and claudin-5, but have also shown a role of systemic inflammatory cytokines including tumor necrosis factor alpha, interleukins IL-1*β* and IL-6 in the pathogenesis of brain edema in experimental ALF, suggesting a vasogenic injury in the blood-brain barrier (BBB) integrity [11–14]. 

## 2. The Structure of Blood-Brain Barrier

The blood-brain barrier is the physical and metabolic barrier separating the peripheral circulation from the central nervous system. The BBB is made up of brain capillary endothelial cells and their junctional complexes [15]. The endothelial cell spreads itself on the basal lamina, covering the entire luminal surface of the capillary with the two surface edges being sealed with junctional molecules forming the tight junction of the BBB. The endothelial cell on the luminal side of the basal lamina, together with the pericyte and astrocytic end foot enveloping the inner face of the basal lamina, and the surrounding neurons collectively represent a neurovascular unit that tightly regulates any exchange between the circulating blood and the central nervous system.

The tight junctions are the major structures responsible for restricting paracellular escape of compounds across the cerebral endothelium. It consists of tight junction proteins including occludin, claudin-5, junctional adhesion molecules (JAM), and cadherins [16]. Tight junction molecules are transmembrane proteins and are associated with the intracellular cytoskeleton via the peripheral junctional molecules zona occludin 1 (ZO-1) and its associates ZO-2 and ZO-3 and catenin isoforms. The tight junction segregates the apical or luminal plasmalemma from the basal or abluminal one, maintaining cellular polarity. The tight junction limits the paracellular diffusion of small molecules, regulating entry of circulating molecules such as water and solutes into the brain parenchyma. The endothelial cell and its tight junction are thus the first barrier of the neurovascular unit. An intact TJ is required to maintain normal BBB function.

**Figure 1:**
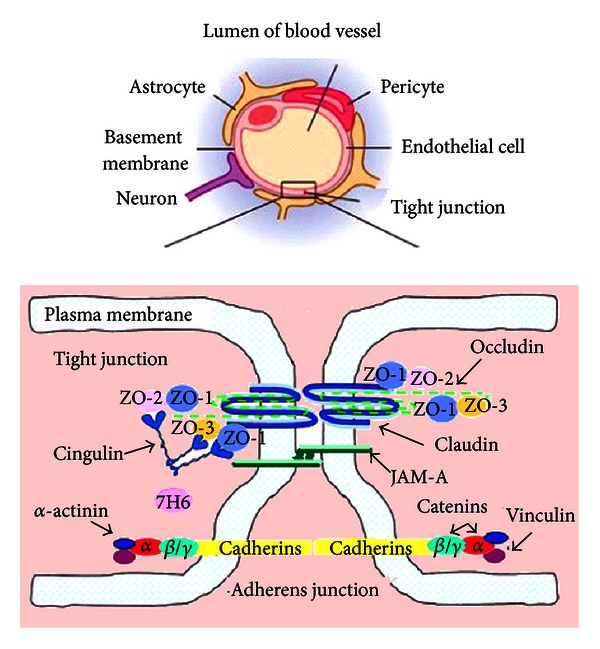
The structure of blood-brain barrier.

Occludin was the first discovered element of the tight junction. It is about 65 kD with C- and N-terminals within the cytoplasm and four membrane-spanning segments. The two extracellular loops participate in the paracellular path [17]. Occludin is expressed in various tissues and organs including epithelial and endothelial layers, especially in brain endothelial cells. Occludin integrity is essential for normal barrier function, whose presence at the BBB is correlated with the high transendothelial electrical resistance across the barrier (1500–2000 Ω∗cm^2^) and decreased paracellular permeability [18]. Therefore, occludin is a reliable marker for tight junction in immunohistochemical evaluation (see Figure 1). 

## 3. Increased BBB Permeability Occurs with Subtle Morphological Modifications of BBB in ALF

In 1977, Livingston and colleagues showed the extravasation of Trypan blue in brains of rats with D-galactosamine-induced ALF [19]. Thereafter, many studies about BBB injury were performed [4, 6, 8, 20, 21]. Most of studies showed subtle alterations of BBB ultrastructure and increased BBB permeability; it is maybe the mechanism of vasogenic brain edema in ALF.

### 3.1. Changes of Morphological Modifications of BBB in ALF

Kato et al. [6] obtained samples of cerebral cortex immediately after death from nine patients with acute liver failure. The ultrastructural appearance of brain capillaries by scanning electron microscopy showed that the intercellular tight junctions between capillary endothelial cells were intact except for being slightly widened in two patients. The endothelial cells were swollen, with increased numbers of vesicles and vacuoles. The basement membranes were enlarged and vacuolized and the pericytes had increased numbers of vesicles and vacuoles. Marked intracellular swelling of the perivascular astroglial foot processes was present. Traber et al. [7] also investigated the swelling of perivascular astroglial foot and the intact tight junctions in rabbits ALF model. The above mentioned results have shown that in subjects with ALF the BBB and its TJ are grossly intact. These findings are consistent with the emerging concept that a vasogenic brain edema results from subtle modifications of TJ proteins without an obvious disruption of the BBB.

**Figure 2:**
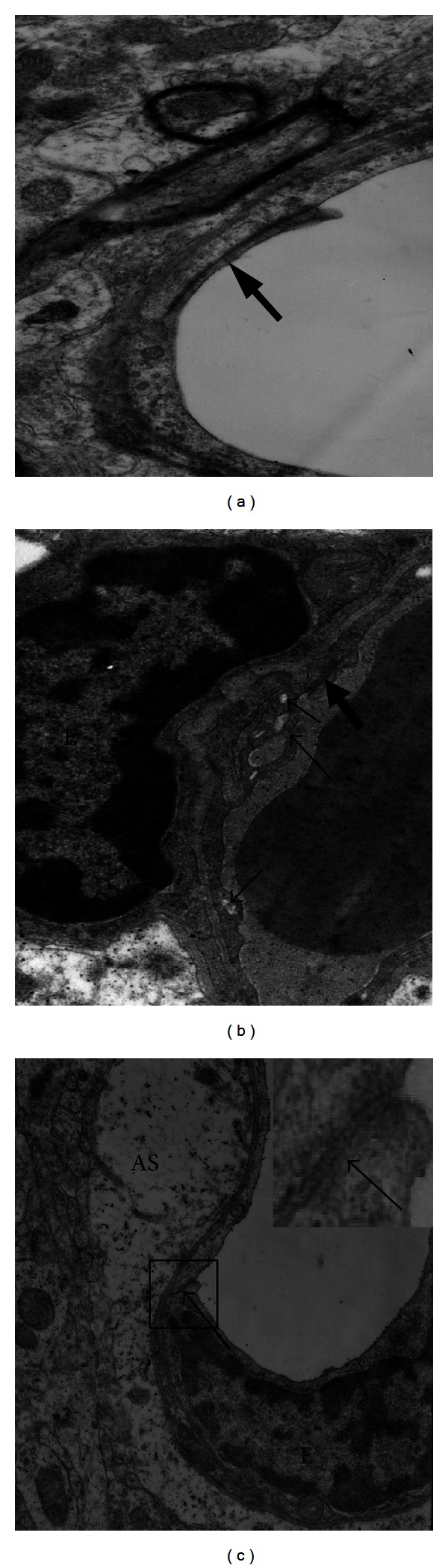
Transmission electron microscopy of mouse brains from D-galactosamine and liposaccharide-induced ALF. (a) Control (×12000 magnification). TJs (arrow) were intact. (b) ALF mouse (×15000 magnification). EC was shrunken, vesicles and vacuoles (small arrows) were present, and TJs (large arrow) were complete. (c) Another ALF mouse (×12000 magnification). The feet of perivascular astroglial cells (AS) were swollen. TJs (arrow) were disrupted. The rectangular area was magnified in the upper right.

We recently report similar alterations in tight junction components in ALF mice induced by D-galactosamine and liposaccharide and in patients' autopsy brain specimen [12]. However, different from other reports, we found that some of the intracellular TJ were disrupted. We attribute the disruption of TJs observed here to differences in methods used to induce liver failure, which could lead to different mechanisms of BBB disruption (see Figure 2).

### 3.2. Studies on BBB Penetration by Different Compounds in ALF

Although many studies reveal only subtle alterations in the BBB of ALF animals and the minimal BBB changes observed in human patients who died of ALF [6], the increased transport of many substrates including large molecules such as horseradish peroxidase (HRP) and small ones such as ammonia and water [22–24] was observed by many studies. The vasogenic edema that resulted from increased BBB permeability is also believed as to be an early step in the development and progression of brain edema in ALF [25]. 

Dixit and Chang in 1990 similarly showed that Trypan blue extravasates into brain parenchyma in rats with ALF-induced by D-galactosamine [25]. The increased permeability to the intravascularly injected dye molecules increased with the degree of hepatic encephalopathy. Ott and Larsen showed that BBB in PCA rats (a model which mimics the condition of portal-systemic shunting in patients with liver cirrhosis) is leaky to HRP [23]. 

These observations have been confirmed by others using different BBB permeability markers and/or hepatic encephalopathy (HE) models [26, 27]. We also found that BBB permeability began to increase approximately two hours after ALF induction in mice, measured by Evans blue (EB) concentration [12], which was in agreement with above-mentioned studies. However, other contemporary animal studies often performed in similar HE models and using similar markers revealed no brain vascular permeability changes [28–30]. It was reckoned that incoherent results in the studies, were due to different chosen times. Controversies about the BBB permeability assessed with different compounds have lasted until the present time [31, 32] (see Table 1).

### 3.3. Transport of Ammonia through BBB in ALF

Ammonia has long been associated with hepatic encephalopathy and brain edema. Arterial ammonia level directly correlates to the risk of impending brain herniation due to brain edema [36, 37]. It has been debated whether blood ammonia enters the brain by passive diffusion and/or active transport by ion-transporters and that changes in blood pH could affect the blood-to-brain transfer of ammonia. Incoherent results were also obtained. Ahl et al. [34] and Lockwood et al. [33] showed that ammonia enters the brain more easily in advanced HE patients than in healthy controls. By contrast Goldbecker et al. [32] did not see any differences in BBB permeability for ammonia between patients with and without liver failure. Sørensen and Keiding [35] observed increased ammonia accumulation in cirrhotic patients, but in their hands the increase was solely attributable to increased blood ammonia content. Different studies results of BBB permeability for ammonia were shown in Table 1.

The conventional assumption is that unionized ammonia (NH_3_) can pass the blood-brain barrier by diffusion, whereas translocation of the ionized species (NH_4_^+^) can be neglected. Since 99% of ammonia exists in ionic form at physiologic pH, it enters the brain mainly by a transcellular route, using an array of potassium channels and transporters or by substituting other cations with similar hydrated radius. How a small polar molecule like NH_4_^+^ traverses BBB in ALF remains incompletely understood. Recently, the paracellular penetration by gaseous ammonia is being taken into consideration as a significant alternative [24]. It is not known whether ammonia transport from blood to brain would increase under excessive ammonia load and increased BBB permeability in ALF.

## 4. Associated Systemic Factors with BBB Permeability in ALF

It is believed that breakdown products of injured and necrotic hepatocytes are released into the systemic circulation. Among the neurotoxic factors, the inflammatory cytokines including tumor necrosis factor, interleukins-1 and -6, interferon, and ammonia are the most important factors [38–40]. Recently, Yamamoto and Nguyen [41] demonstrated that matrix metalloproteinase-9 induces significant alterations in TJ proteins resulting in increased permeability and brain edema. 

### 4.1. Tumor Necrosis Factor Alpha

Inflammatory molecules, including cytokines interleukin-1 (IL-1), interleukin-6 (IL-6), and tumor necrosis factor-alpha (TNF-*α*), are increased in plasma during acute and chronic liver failure in patients [42] and in animals with experimentally induced HE [39]. Circulating levels of TNF-*α* correlate positively with the severity of HE [43] and with the intracranial pressure in ALF patients [44]. 

Tumor necrosis factor alpha has been shown to play a crucial role in the regulation of BBB permeability in human patients and animals with ALF. Selective deletion of cellular receptors of interleukin-1 and tumor necrosis factor alpha attenuates the development of encephalopathy and brain edema in experimental ALF [38]. Injection of TNF-*α* into the central nervous system could increase the permeability of BBB, which was TNF-*α*-dose dependent. And the anti-TNF-*α* antibody could inhibit the increase of the BBB permeability and block the entering of bacterium into the CNS [45, 46].

And in our study, we also focused on the role of TNF-*α* in changing the permeability of BBB during ALF [12, 31]. We treated the ALF mice induced by D-galactosamine and lipopolysaccharide or acetaminophen with TNF-*α*, anti-TNF-*α*, or anti-TNF-*α*-R1 antibody previous administration to block the ALF induction. Then, subjects concerning to the BBB permeability including BBB ultrastructure, EB passage into brain tissue, and the expression of tight junction protein occludin were measured. We found that EB concentration in brain was significantly increased in mice injected with GalN/LPS as well as with TNF-*α* alone. However, in animals pretreated with either of the two anti-TNF-*α* antibodies, the concentration of EB in brain tissue significantly decreased compared with GalN/LPS mice. Furthermore, tight junction protein occludin and its accessory associates ZO-1 were significantly decreased in brains of ALF mice [12, 31], which were also blocked by TNF-*α* antibody or TNF-*α*-R1 antibody. These results further confirmed that the enhanced production of TNF-*α* induced by GalN/LPS was responsible for the tight junction protein disruption that resulted in apparent increase of BBB permeability in ALF. 

Changes in BBB permeability and structure may be caused directly by TNF-*α* or indirectly through other mediators released by the necrotic liver, such as matrix metalloproteinase-9, NO, and free radicals [14, 47]. The signal mechanism of TNF-*α* that increase the permeability of BBB was investigated further. Chen et al. and Aslam et al. reported that TNF-*α* acts through TLR4/NF*κ*B signaling for the down-regulation of tight junction protein expression [48, 49]. Defazio et al. observed that TNF-*α* upregulated intercellular adhesion molecule 1 (ICAM1) expression and fluid phase endocytosis (FPE) of horseradish peroxidase through kinase C (PKC) pathway, not through protein kinase A on brain microvascular endothelial cell (BMEC) culture [50].

### 4.2. Matrix Metalloproteinase-9

MMP-9 plays an important role in numerous physiologic and pathologic processes. Specifically, MMP-9 can digest capillary endothelial constituents and TJ proteins of BBB. Recent studies revealed that brain edema in azoxymethane-induced ALF, was found to be secondary to tight junction protein degradation mediated by activation of MMP-9 [40]. Specifically, it has been shown that TJ proteins occludin and claudin-5 are significantly degraded in the brains of mice with galactosamine-induced ALF, and this effect was reversed by treatment with inhibitor of MMP-9, GM6001 [11]. Laursen et al. [22] also observed that progression of intracranial pressure in the course of ALF is strictly correlated with the increase in BBB permeability and MMP-9 content. Basing on this study the authors proposed a sequence of events of ALF-induced brain damage, in which increase in BBB permeability is an initial step leading to vasogenic edema followed by ammonia excitotoxicity and cytotoxic edema.

The most likely sequence of MMP-9 to occludin degradation in ALF mice was delineated by Chen et al. [51, 52]; in this study, the intermediate steps include transactivation of epidermal growth factor receptor (EGFR) and p38 MAPK/NF*κ*B (mitogen-activated protein kinase/nuclear factor kappa B). 

### 4.3. Ammonia

**Figure 3:**
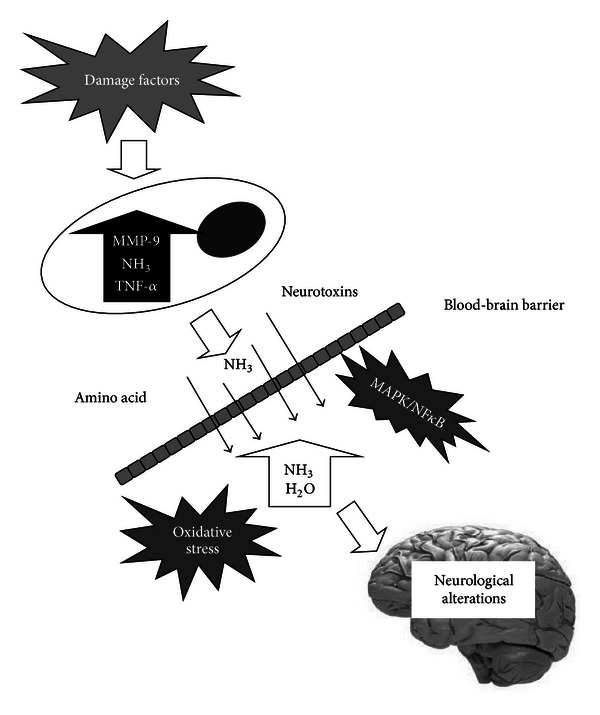
The pathogenesis of brain edema in ALF involves the action of neurotoxins such as ammonia and TNF-*α* and MMP-9, as well as various phenomena that include alterations in neurotransmission, blood-brain barrier permeability, and energy metabolism.

Apart from directly affecting the metabolism and function of the central nervous system cells, ammonia influences the passage of different molecules across the blood-brain barrier (BBB). Hyperammonemia was shown to be directly responsible for PCA-induced alterations in the metabolism and transport of amino acids [53]. Recently, Skowrońska et al. [54] confirmed the role of ammonia on the vasogenic mechanism to the cerebral edema. They found that treatment of a rat brain endothelial cell line (RBE-4) with ammonia (5 mmol/L, 24 h) increased cell permeability to fluorescein isothiocyanate (FITC)-dextran (40 kDa). Concurrently, ammonia increased the activity of extracellular matrix metalloproteinases (MMP-2/MMP-9) and caused accumulation of ONS markers such as reactive oxygen species (ROS), nitric oxide (NO), and peroxidation products of phospholipid-bound arachidonic acid, F2-isoprostanes (F2-IsoPs). The increase of cell permeability was ameliorated upon cotreatment with a MMP inhibitor, SB-3CT, and with an antioxidant, glutathione diethyl ester (GEE). These results support the concept that ammonia increases paracellular permeability of BBB by a mechanism encompassing oxidative/nitrosative stress and activation of matrix metalloproteinases in ALF (see Figure 3).

## 5. Summary

Brain edema is a severe complication in ALF. Recent studies implicate that the vasogenic pathway is the leading event in the pathogenesis of brain edema in ALF. It is associated with BBB dysfunction without overt structural breakdown. It had been approved by many researches. One of the long disputed issues is that whether permeability of BBB to ammonia was increased in cerebral edema of ALF. Some studies found that both regional and total cerebral blood flow (CBF) were significantly reduced in cirrhosis patients with HE when compared to cirrhosis patients without HE and healthy subjects [55] and when compared to after recovery from HE [56]. Therefore, it was thought that increased ammonia level in brain of patients with cirrhosis did not certify any real changes in the permeability for ammonia [57]. This point of view did not apply to ALF, when the rise of CBF occurred in ALF.

Inflammatory molecule-TNF-*α* and MMP-9 have an obvious effect on TJ protein degradation that resulted in BBB permeability damage, which was involved with MAPK/NF*κ*B pathway. Underlying mechanism needed further studies. The above described results will be useful for the further investigation of the molecular pathogenesis of brain edema and may potentially lead to effective therapeutic interventions for the prevention of brain edema in ALF.

## Figures and Tables

**Table 1:** Different studies on BBB penetration by different compounds in ALF.

Study	ALF model	Tracer for BBB permeability	Results
Dixit and Chang [24]	D-galactosamine induced ALF rats	Trypan blue	+
Laursen et al. [22]	PCA rats	HRP	+
Zaki et al. [26]	PCA rats	Amino acid	+
Horowitz et al. [27]	Galactosamine-induced ALF rats	Aminoisobutyric acid	+
Lv et al. [12]	D-galactosamine/liposaccharide	Evans blue	+
Chavarria et al. [28]	PCA rats	^ 14^C-labeled sucrose	−
Sarna et al. [29]	PCA rats	Amino acid	−
Alexander et al. [30]	Portacaval shunting rats	Labelled mannitol and amino acids	−
Ahl et al. [33]	Patients	Ammonia	+
Lockwood et al. [34]	Patients	Ammonia	+
Sørensen and Keiding [35]	Patients	Ammonia	+
Goldbecker et al. [32]	Patients	Ammonia	−

PCA: a model which mimics the condition of portal-systemic shunting in patients with liver cirrhosis.

## References

[1] Lee WM, Squires RH, Nyberg SL, Doo E, Hoofnagle JH (2008). Acute liver failure: summary of a workshop. *Hepatology*.

[2] Vaquero J, Blei AT (2003). Etiology and management of fulminant hepatic failure. *Current Gastroenterology Reports*.

[3] Shawcross DL, Wendon JA (2012). The neurological manifestations of acute liver failure. *Neurochemistry International*.

[4] Kimelberg HK (2004). Water homeostasis in the brain: basic concepts. *Neuroscience*.

[5] Klatzo I (1967). Neuropathological aspects of brain edema. *Journal of Neuropathology and Experimental Neurology*.

[6] Kato M, Hughes RD, Keays RT, Williams R (1992). Electron microscopic study of brain capillaries in cerebral edema from fulminant hepatic failure. *Hepatology*.

[7] Traber PG, Canto MD, Ganger DR, Blei AT (1987). Electron microscopic evaluation of brain edema in rabbits with galactosamine-induced fulminant hepatic failure: ultrastructure and integrity of the blood-brain barrier. *Hepatology*.

[8] Gove CD, Hughes RD, Ede RJ, Williams R (1997). Regional cerebral edema and chloride space in galactosamine-induced liver failure in rats. *Hepatology*.

[9] Nguyen JH (2012). Blood-brain barrier in acute liver failure. *Neurochemistry International*.

[10] Wright G, Sharifi Y, Jalan R (2010). Blood-brain barrier in liver failure: are cracks appearing in the wall?. *Liver International*.

[11] Chen F, Ohashi N, Li W, Eckman C, Nguyen JH (2009). Disruptions of occludin and claudin-5 in brain endothelial cells in vitro and in brains of mice with acute liver failure. *Hepatology*.

[12] Lv S, Song HL, Zhou Y (2010). Tumour necrosis factor-*α* affects blood-brain barrier permeability and tight junction-associated occludin in acute liver failure. *Liver International*.

[13] Skowrońska M, Zielińska M, Wójcik-Stanaszek L (2012). Ammonia increases paracellular permeability of rat brain endothelial cells by a mechanism encompassing oxidative/nitrosative stress and activation of matrix metalloproteinases. *Journal of Neurochemistry*.

[14] Nguyen JH, Yamamoto S, Steers J (2006). Matrix metalloproteinase-9 contributes to brain extravasation and edema in fulminant hepatic failure mice. *Journal of Hepatology*.

[15] Abbott NJ, Patabendige AAK, Dolman DEM, Yusof SR, Begley DJ (2010). Structure and function of the blood-brain barrier. *Neurobiology of Disease*.

[16] Vorbrodt AW, Dobrogowska DH (2003). Molecular anatomy of intercellular junctions in brain endothelial and epithelial barriers: electron microscopist’s view. *Brain Research Reviews*.

[17] Liu WY, Wang ZB, Zhang LC, Wei X, Li L (2012). Tight junction in blood-brain barrier: an overview of structure, regulation, and regulator substances. *CNS Neuroscience & Therapeutics*.

[18] Sandoval KE, Witt KA (2008). Blood-brain barrier tight junction permeability and ischemic stroke. *Neurobiology of Disease*.

[19] Livingstone AS, Potvin M, Goresky CA (1977). Changes in the blood-brain barrier in hepatic coma after hepatectomy in the rat. *Gastroenterology*.

[20] Kristiansen RG, Lindal S, Myreng K (2010). Neuropathological changes in the brain of pigs with acute liver failure. *Scandinavian Journal of Gastroenterology*.

[21] Thumburu KK, Taneja S, Vasishta RK, Dhiman RK (2012). Neuropathology of acute liver failure. *Neurochemistry International*.

[22] Laursen H, Schrøder H, Westergaard E (1975). The effect of portocaval anastomosis on the permeability to horseradish peroxidase of cerebral vessels of the rat. *Acta Pathologica Microbiologica Scandinavica A*.

[23] Ott P, Larsen FS (2004). Blood-brain barrier permeability to ammonia in liver failure: a critical reappraisal. *Neurochemistry International*.

[24] Dixit V, Chang TMS (1990). Brain edema and the blood brain barrier in galactosamine-induced fulminant hepatic failure rats. An animal model for evaluation of liver support systems. *ASAIO Transactions*.

[25] Cauli O, López-Larrubia P, Rodrigo R (2011). Brain region-selective mechanisms contribute to the progression of cerebral alterations in acute liver failure in rats. *Gastroenterology*.

[26] Zaki AEO, Wardle EN, Canalese J (1983). Potential toxins of acute liver failure and their effects on blood-brain barrier permeability. *Experientia*.

[27] Horowitz ME, Schafer DF, Molnar P (1983). Increased blood-brain transfer in a rabbit model of acute liver failure. *Gastroenterology*.

[28] Chavarria L, Oria M, Romero-Gimenez J, Alonso J, Lope-Piedrafita S, Cordoba J (2010). Diffusion tensor imaging supports the cytotoxic origin of brain edema in a rat model of acute liver failure. *Gastroenterology*.

[29] Sarna GS, Bradbury MWB, Cavanagh J (1977). Permeability of the blood-brain barrier after portocaval anastomosis in the rat. *Brain Research*.

[30] Alexander B, Li X, Benjamin IS, Segal MB, Sherwood R, Preston JE (2000). A quantitative evaluation of the permeability of the blood brain barrier of portacaval shunted rats. *Metabolic Brain Disease*.

[31] Wang W, Lv S, Zhou Y, Fu J, Li C, Liu P (2011). Tumor necrosis factor-*α* affects blood-brain barrier permeability in acetaminophen-induced acute liver failure. *European Journal of Gastroenterology and Hepatology*.

[32] Goldbecker A, Buchert R, Berding G (2010). Blood-brain barrier permeability for ammonia in patients with different grades of liver fibrosis is not different from healthy controls. *Journal of Cerebral Blood Flow and Metabolism*.

[33] Ahl B, Weissenborn K, van den Hoff J (2004). Regional differences in cerebral blood flow and cerebral ammonia metabolism in patients with cirrhosis. *Hepatology*.

[34] Lockwood AH, Yap EWH, Wong WH (1991). Cerebral ammonia metabolism in patients with severe liver disease and minimal hepatic encephalopathy. *Journal of Cerebral Blood Flow and Metabolism*.

[35] Sørensen M, Keiding S (2007). New findings on cerebral ammonia uptake in HE using functional ^13^N-ammonia PET. *Metabolic Brain Disease*.

[36] Bhatia V, Singh R, Acharya SK (2006). Predictive value of arterial ammonia for complications and outcome in acute liver failure. *Gut*.

[37] Zwirner K, Thiel C, Thiel K, Morgalla MH, Königsrainer A, Schenk M (2010). Extracellular brain ammonia levels in association with arterial ammonia, intracranial pressure and the use of albumin dialysis devices in pigs with acute liver failure. *Metabolic Brain Disease*.

[38] Bémeur C, Qu H, Desjardins P, Butterworth RF (2010). IL-1 or TNF receptor gene deletion delays onset of encephalopathy and attenuates brain edema in experimental acute liver failure. *Neurochemistry International*.

[39] Jiang W, Desjardins P, Butterworth RF (2009). Direct evidence for central proinflammatory mechanisms in rats with experimental acute liver failure: protective effect of hypothermia. *Journal of Cerebral Blood Flow and Metabolism*.

[40] Skowrońska M, Albrecht J (2012). Alterations of blood brain barrier function in hyperammonemia: an overview. *Neurotoxicity Research*.

[41] Yamamoto S, Nguyen JH (2006). TIMP-1/MMP-9 imbalance in brain edema in rats with fulminant hepatic failure. *Journal of Surgical Research*.

[42] Wright G, Shawcross D, Olde Damink SWM (2007). Brain cytokine flux in acute liver failure and its relationship with intracranial hypertension. *Metabolic Brain Disease*.

[43] Odeh M, Sabo E, Srugo I, Oliven A (2005). Relationship between tumor necrosis factor-alpha and ammonia in patients with hepatic encephalopathy due to chronic liver failure. *Annals of Medicine*.

[44] Jalan R, Olde Damink SW, Hayes PC, Deutz NEP, Lee A (2004). Pathogenesis of intracranial hypertension in acute liver failure: inflammation, ammonia and cerebral blood flow. *Journal of Hepatology*.

[45] Tsao N, Hsu HP, Wu CM, Liu CC, Lei HY (2001). Tumour necrosis factor-*α* causes an increase in blood-brain barrier permeability during sepsis. *Journal of Medical Microbiology*.

[46] Ábrahám CS, Deli MA, Joó F, Megyeri P, Torpier G (1996). Intracarotid tumor necrosis factor-*α* administration increases the blood-brain barrier permeability in cerebral cortex of the newborn pig: quantitative aspects of double-labelling studies and confocal laser scanning analysis. *Neuroscience Letters*.

[47] Mayhan WG (2002). Cellular mechanisms by which tumor necrosis factor-*α* produces disruption of the blood-brain barrier. *Brain Research*.

[48] Chen G, Zhang S, Shi J, Ai J, Qi M, Hang C (2009). Simvastatin reduces secondary brain injury caused by cortical contusion in rats: possible involvement of TLR4/NF-*κ*B pathway. *Experimental Neurology*.

[49] Aslam M, Ahmad N, Srivastava R, Hemmer B (2012). TNF-alpha induced NF*κ*B signaling and p65 (RelA) overexpression repress Cldn5 promoter in mouse brain endothelial cells. *Cytokine*.

[50] Defazio G, Nico B, Trojano M (2000). Inhibition of protein kinase C counteracts TNF*α*-induced intercellular adhesion molecule 1 expression and fluid phase endocytosis on brain microvascular endothelial cells. *Brain Research*.

[51] Chen F, Hori T, Ohashi N, Baine AM, Eckman CB, Nguyen JH (2011). Occludin is regulated by epidermal growth factor receptor activation in brain endothelial cells and brains of mice with acute liver failure. *Hepatology*.

[52] Chen F, Radisky ES, Das P (2013). TIMP-1 attenuates blood-brain barrier permeability in mice with acute liver failure. *Journal of Cerebral Blood Flow & Metabolism*.

[53] Jessy J, Mans AM, DeJoseph MR, Hawkins RA (1990). Hyperam-monaemia causes many of the changes found after portacaval shunting. *Biochemical Journal*.

[54] Skowrońska M, Zielińska M, Wójcik-Stanaszek L (2012). Ammonia increases paracellular permeability of rat brain endothelial cells by a mechanism encompassing oxidative/nitrosative stress and activation of matrix metalloproteinases. *Journal of Neurochemistry*.

[55] Iversen P, Sørensen M, Bak LK (2009). Low cerebral oxygen consumption and blood flow in patients with cirrhosis and an scute episode of hepatic encephalopathy. *Gastroenterology*.

[56] Dam G, Keiding S, Munk OL (2013). Hepatic encephalopathy is associated with decreased cerebral oxygen metabolism and blood flow, not increased ammonia uptake. *Hepatology*.

[57] Sørensen M (2013). Update on cerebral uptake of blood ammonia. *Metabolic Brain Disease*.

